# A Case of Cryoglobulinemic Nephritis That Responded to Rituximab Monotherapy

**DOI:** 10.7759/cureus.85308

**Published:** 2025-06-03

**Authors:** Kumiko Fujieda, Akihito Tanaka, Kayaho Maeda, Takaya Ozeki, Kazuhiro Furuhashi, Shoichi Maruyama

**Affiliations:** 1 Department of Nephrology, Nagoya University Hospital, Nagoya, JPN; 2 Department of Nephrology, Nagoya University Graduate School of Medicine, Nagoya, JPN

**Keywords:** cryoglobulinemia, cryoglobulinemic nephritis, cryoglobulinemic vasculitis, nephritis, rituximab, rituximab monotherapy

## Abstract

The patient was a 65-year-old woman with ascites effusion and portal hypertension of unknown cause for the past three years, who was followed up with diuretics. Subsequently, the patient developed abdominal distention and worsening leg edema, and the laboratory test results revealed positive antinuclear antibodies, mild renal impairment, and abnormal urinalysis. After renal biopsy and laboratory tests, the patient was diagnosed with cryoglobulinemic nephritis. She was treated with rituximab monotherapy, which resulted in decreased serum creatinine and urinary protein levels and suppression of complement lowering. Thus, rituximab treatment may be effective for cryoglobulinemic nephritis.

## Introduction

Cryoglobulinemic vasculitis arises when cryoglobulins - immunoglobulins (Igs) that reversibly precipitate at temperatures below 37°C and redissolve upon warming - aggregate at low temperatures, provoking systemic small-artery vasculitis and resulting in lesions of the skin, peripheral nerves, and kidneys [1]. The precipitation of cryoglobulins under cold conditions induces immune complex formation and complement activation, which drive inflammation and vascular damage [1]. Clinically, it presents as a systemic disease with features such as purpura, arthralgia, and general fatigue - collectively known as Meltzer’s triad - as well as possible involvement of peripheral nerves and kidneys [1]. Laboratory findings may also reveal low complement levels and the presence of circulating cryoglobulins.

It is classified as types I-III based on the Ig deposition [2-4]. Type I is characterized by deposits of monoclonal Ig and accounts for 5-25% of cryoglobulinemic vasculitis cases, with background diseases such as monoclonal gammopathy of undetermined significance and B-cell malignancies [2-4]. Type II, in which both polyclonal and monoclonal Ig are deposited, accounts for approximately 50% of cryoglobulinemic vasculitis cases. Background diseases associated with type II cryoglobulinemic vasculitis include hepatitis C virus (HCV) infection, hepatitis B virus (HBV) infection, human immunodeficiency virus infection, autoimmune diseases, and lymphoproliferative disorders [2-4]. Type III, in which polyclonal Ig is deposited, accounts for approximately 40% of cryoglobulinemic vasculitis cases. Background diseases associated with type III cryoglobulinemic vasculitis include HCV infections and autoimmune diseases [2-4]. Types II and III together are defined as mixed cryoglobulinemic vasculitis. Historically, chronic HCV infection was strongly associated with mixed cryoglobulinemia and represented its leading cause. However, with the advent of direct-acting antivirals, the number of HCV-related cases has been declining in recent years [5]. The classification, epidemiology, and clinical associations of cryoglobulinemia are shown in the Appendix.

Cryoglobulinemic glomerulonephritis is reported to occur in approximately 20-30% of patients with mixed cryoglobulinemic vasculitis [6]. Clinically, it typically presents with hematuria, proteinuria, hypertension, and varying degrees of renal dysfunction [7]. Histopathologically, it is most commonly characterized by membranoproliferative glomerulonephritis [7]. Immunofluorescence typically reveals granular deposition of IgM, IgG, and complement components [7]. Electron microscopy may show organized or microtubular deposits, reflecting cryoglobulin precipitation [7]. When renal involvement is present, the prognosis is generally poor [3], necessitating aggressive therapeutic intervention. However, corticosteroids may be given concurrently with the treatment of underlying causes, with a great risk of complications such as infection, and may lead to a decline in activities of daily living (ADL), particularly in elderly patients. Therefore, balancing the immunosuppressive efficacy with the risk of adverse effects remains a clinical challenge. Therefore, it is crucial to explore effective alternatives with safer profiles.

Here, we report a case of cryoglobulinemic glomerulonephritis successfully treated with rituximab without the use of corticosteroids. This case highlights a potentially safer and effective therapeutic approach for managing renal involvement in cryoglobulinemic vasculitis, particularly in elderly or frail populations where conventional immunosuppression poses substantial risk.

## Case presentation

**Figure 1:**
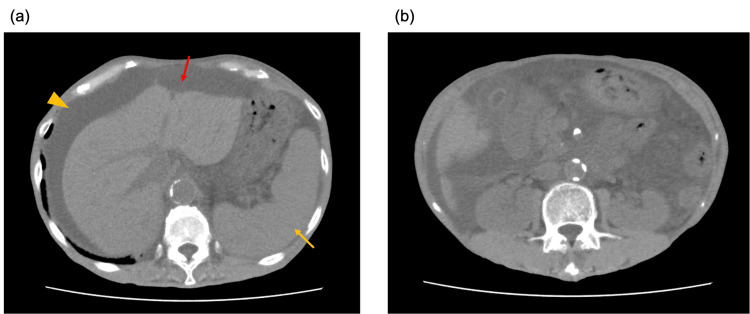
Abdominal computed tomography (a) Deformed liver (suggestive of chronic liver damage; the red arrow), ascites (the yellow arrowhead), and splenomegaly (the yellow arrow). (b) No obvious renal atrophy is observed.

The patient was a 65-year-old woman who had visited her previous physician three years earlier with a chief complaint of abdominal pain. She was found to have ascites and was diagnosed with portal hypertension. The patient did not have cirrhosis and tested negative for hepatitis B virus (HBV) and hepatitis C virus (HCV). Liver biopsy findings showed some bridging fibrosis, but it was limited to some portal areas and was not considered suggestive of liver cirrhosis. Inflammatory cell infiltration was minimal, with only a mild lymphocytic infiltrate in the portal area. Therefore, the ascites was considered to be due to portal hypertension, not caused by cirrhosis or autoimmune hepatitis. Portal hypertension was thought to be due to an arteriovenous malformation of the liver, but the cause could not be determined. Ascites was controlled with 20 mg/d furosemide and 25 mg/d spironolactone. The following month, prophylactic endoscopic variceal ligation was performed for esophageal varices. Six months earlier, the dose of diuretics was increased to 40 mg/d furosemide and 50 mg/d spironolactone, owing to worsening abdominal distention. Computed tomography revealed ascites, liver deformation suggestive of chronic liver damage, and splenomegaly (Fig. 1). The blood test performed for screening revealed negative results for anti-mitochondrial antibodies and anti-smooth muscle antibodies, but positive results for antinuclear antibodies at 1:640. In addition, the patient showed mild renal dysfunction with a serum creatinine (Cr) level of 1.26 mg/dL and abnormal urinalysis with urine protein (1+) and urine occult blood (3+); therefore, the patient was referred to the department of nephrology for a consultation. Other preexisting medical conditions included hypertension, dyslipidemia, rheumatoid arthritis, pancreatic body intraductal papillary mucinous neoplasm, uterine myoma, and emphysema. The medications included furosemide (40 mg/d) and spironolactone (50 mg/d) for ascites; esomeprazole magnesium hydrate (20 mg/d) for gastrointestinal protection; rosuvastatin calcium (2.5 mg/d) for dyslipidemia; irbesartan (100 mg/d) and amlodipine besylate (5 mg/d) for hypertension; pantethine (300 mg/d) and magnesium oxide (990 mg/d) for constipation; and methotrexate (12 mg/week), folic acid (5 mg/d), and iguratimod (50 mg/d) for rheumatoid arthritis. She was 158 cm tall, weighed 43 kg, and had clear consciousness.

**Figure 2:**
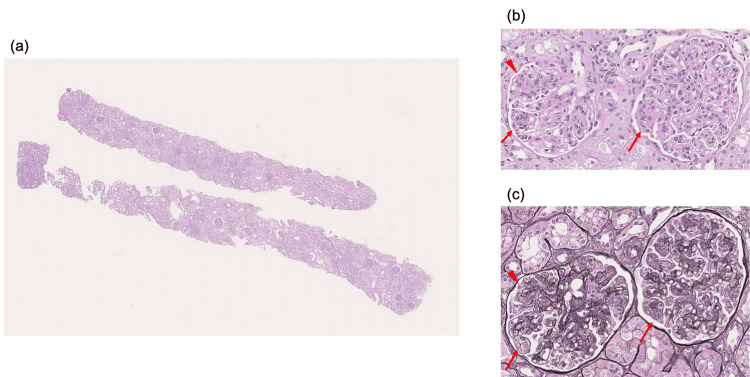
Light microscopy findings of the renal biopsy specimen Diffusely, intraductal cell proliferation, including that of neutrophils, is prominent (the red arrows); mesangiolysis is observed in areas of severe inflammation (the red arrowhead). Segmentalization was also observed in some glomeruli. (a)  Periodic acid-Schiff staining, ×100; (b) periodic acid-Schiff staining, ×400; (c) periodic acid methenamine silver staining, ×400.

**Figure 3:**
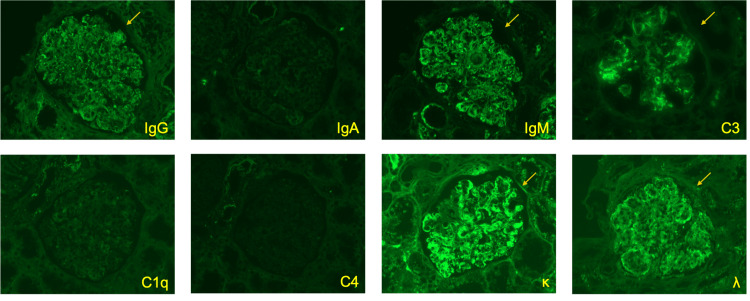
Immunofluorescence analysis of glomeruli Immunofluorescence analysis was performed using fluorescein-isothiocyanate-conjugated antibodies against immunoglobulin (Ig)G, IgA, IgM, C3, C1q, C4, κ, and λ; glomerular staining reveals granule positivity for IgG, IgM, C3, κ, and λ in the mesangial area (the yellow arrows) and partial capillary, while IgA, C1q, and C4 are all negative.

**Figure 4:**
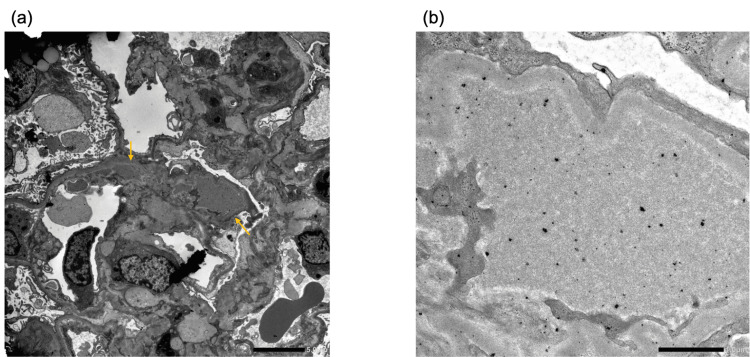
Electron microscopic analysis of glomeruli (a) Electron microscopy reveals electron-dense deposits in the mesangial areas and subendothelial space (arrows) (scale bar, 5.0 μm). (b) No tubular structures characteristic of cryoglobulinemia are observed (scale bar, 1.0 μm).

Vital signs on arrival were as follows: body temperature, 37.2℃; blood pressure, 93/49 mmHg; pulse, 84 beats/min; and SpO_2_, 98% (room air). Physical examination revealed abdominal distention and petechial telangiectasias of the extremities. There was no evidence of leg edema, arthralgia, or peripheral neuropathy. The blood test results at the time of the hospital visit are shown in Table 1, and the urinalysis results are shown in Table 2. The cryoglobulin qualitative test result was borderline positive, and the cryoglobulin immunoelectrophoresis test showed IgM-κ monoclonal (M) protein, which led to suspicion of cryoglobulinemia. Renal biopsy was performed on the 28th day of the illness for diagnosis. Light microscopy revealed 26 glomeruli and one total sclerosing glomerulus (Fig. 2). The glomeruli exhibited a diffuse membranoproliferative pattern of glomerulonephritis. Intraductal cell proliferation, including that of neutrophils, was observed in nine glomeruli, and mesangiolysis was observed in three glomeruli at the site of severe inflammation. No crescent bodies were observed. Segmentalization was also observed in some glomeruli. No evidence of arterial vasculitis was found. Fluorescence imaging revealed granule positivity for IgG (+), IgM (2+), C3 (2+), κ (2+), and λ(+) in the mesangial area and partial capillary, while IgA, C1q, and C4 are all negative (Fig. 3). Electron microscopy revealed electron-dense deposits in some mesangial areas; however, no tubular structures characteristic of cryoglobulinemic nephritis were observed (Fig. 4). The pathological results were not typical of cryoglobulinemic nephritis; therefore, a close examination for hematologic disease was performed. Bone marrow clot analysis revealed bone marrow tissue with prominent collagen-like degeneration and mild hypoplasia, with cellularity of 20-30%. Immunostaining results showed that the cluster of differentiation (CD) 138-positive plasma cells accounted for 5-10% of bone marrow cells, and clusters of these cells were present. Light chain restriction was not evident on κ and λ staining. Serum protein electrophoresis did not reveal M-peaks. Bone marrow droplet digital polymerase chain reaction yielded negative results for the myeloid differentiation primary response gene (Myd) 88 mutation. Bone marrow flow cytometry revealed no surface features suggestive of primary macroglobulinemia or lymphoplasmacytic lymphoma. M-protein was detected, and immunofluorescence revealed a difference in staining intensity between κ and λ light chains. Accordingly, a detailed evaluation for monoclonal gammopathy, including macroglobulinemia and lymphoma, was performed; however, no definitive hematologic malignancy was identified. Furthermore, previous studies have reported that characteristic organized deposits on electron microscopy are observed in approximately 76% of cryoglobulinemic nephritis cases [7], indicating that such findings are not always present. Based on the clinical, laboratory, and pathological findings, the patient was diagnosed with cryoglobulinemic nephritis.

**Table 1:** Result of the blood test on the first visit PT-INR, prothrombin time-international normalized ratio; APTT, activated partial thromboplastin time; Ig, immunoglobulin; CH50, homolytic complement activity; C3, complement C3; C4, complement C4; MMP, matrix metalloprotease; PR3, proteinase 3; ANCA, antineutrophil cytoplasmic antibodies; MPO, myeloperoxidase; GBM, glomerular basement membrane; ds, double strand; ss, single strand; HBs, hepatitis B surface; HCV, hepatitis C virus; TP, treponema pallidum; RPR, rapid plasma reagin

Parameter	Patient values	Normal values
White blood cell count (/μl)	2900	3300-8600
Red blood cell count (10^6^/μl)	2.5	3.86-4.92
Hemoglobin (g/dl)	7.4	11.6-14.8
Platelet count (10^4^/μl)	17.5	15.8-34.8
PT-INR	1.00	
APTT ratio	1.1	
Glucose (mg/dL)	119	73-109
Blood urea nitrogen (mg/dL)	18.2	8.0-20.0
Creatinine (mg/dL)	1.26	0.46-0.79
Sodium (mmol/L)	141	138-145
Potassium (mmol/L)	3.1	3.6-4.8
Chlorine (mmol/L)	103	101-108
Calcium (mg/dL)	8.9	8.8-10.1
Total protein (g/dL)	7.3	6.6-8.1
Albumin (g/dL)	3.1	4.1-5.1
Aspartate aminotransferase (U/L)	33	13-30
Alanine aminotransferase (U/L)	30	7-23
γ-glutamyl transpeptidase (U/L)	52	9-32
Total bilirubin (mg/dL)	1.0	0.4-1.5
Lactate dehydrogenase (U/L)	330	124-222
Alkaline phosphatase (U/L)	176	38-113
C-reactive protein (mg/dL)	5.58	<0.14
IgG (mg/dL)	1302	861-1747
IgA (mg/dL)	143	93-393
IgM (mg/dL)	536	50-269
CH50 (U/mL)	13.7	31.6-57.6
C3 (mg/dL)	63.3	73-138
C4 (mg/dL)	1.5	11-31
Rheumatoid factor (IU/mL)	95.2	<15
MMP-3 (ng/mL)	99.1	17.3-59.7
PR3-ANCA (U/mL)	0	<3.5
MPO-ANCA (U/mL)	0	<3.5
Anti-GBM antibody (U/mL)	0	<3.0
Anti-nuclear antibodies	640 (speckled)	<40
ds-DNAantibody (U/mL)	5.2	<10.0
ss-DNA antibody (U/mL)	14.7	<7.0
Cryoglobulin qualitative test	false positive	
Cryoglobulin immunoelectrophoresis	M protein of IgM-κ	
HBs-antigen	negative	
HBs-antibody	negative	
HCV-antibody	negative	
Anti-TP antibody	negative	
RPR test	negative	

**Table 2:** Result of the urine test on the first visit Cr, creatinine; HPF, high-power field

Urinalysis	Patient values
Proteinuria	1+
Protein	0.3 g/gCr
Occult blood	3+
Red blood cells	50/HPF
Dysmorphic red blood cells	Moderate
White blood cells	10-19/HPF
Bacteria	1+

**Figure 5:**
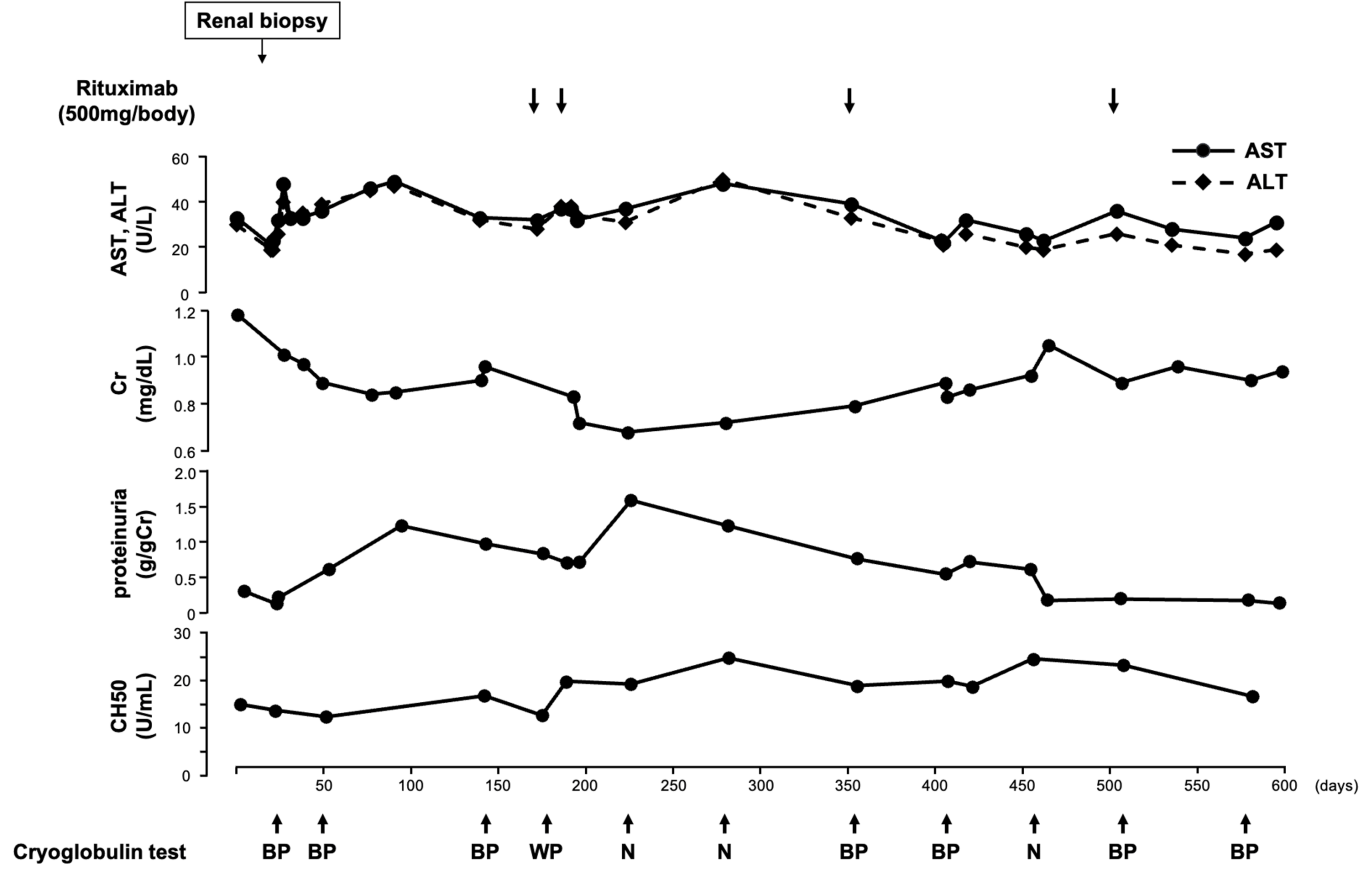
Clinical course Changes in creatinine, proteinuria, and hemolytic complement activity. Rituximab was initiated on the 175th day of the illness, with a second dose administered two weeks apart, followed by a total of four doses of rituximab approximately every six months (arrows). BP, borderline positive; WP, weak positive; N, negative; Cr, creatinine; CH50, hemolytic complement activity

The patient was older, had rheumatoid arthritis, and was frail; therefore, we determined that she could not tolerate treatment with more than moderate doses of steroids, and she was followed up. However, during follow-up, an increase in proteinuria was observed. The patient had already been receiving immunosuppressive agents such as methotrexate and iguratimod for rheumatoid arthritis. Therefore, we determined that adding corticosteroids would further increase the risk of steroid-related complications, including infections and muscle weakness. Given these considerations, we opted for rituximab monotherapy without concomitant steroid use. Because the drug was not covered by insurance, an ethics application was submitted and approved for off-label use (approval number: TT23064). We administered rituximab at a dose of 500 mg/body, which is the approved dosage for nephrotic syndrome in Japan. Rituximab monotherapy was initiated on the 175th day of the illness, with a second dose administered two weeks apart, followed by a total of four doses of rituximab approximately every six months (Fig. 5). A decrease in serum Cr and urinary protein levels was observed. In addition, the reduction in complement was suppressed. The changes in CH50 levels are shown in Fig. 5. The initial C4 level was low at 1.5 mg/dL, but after starting treatment, it also increased and remained around 2-3 mg/dL. The C3 level remained within the range of 50-70 mg/dL. No adverse events were observed during the course of the study, and the patient's ADL did not deteriorate.

## Discussion

The patient had a history of rheumatoid arthritis and a strongly positive rheumatoid factor. Laboratory tests revealed markedly low C3 levels, and kidney biopsy showed deposits of both IgG and IgM. These findings led to the diagnosis of mixed cryoglobulinemia (type Ⅱ), and she was treated with rituximab monotherapy. In this case, tubular structures characteristic of cryoglobulinemic nephritis were not observed on electron microscopy images; nevertheless, there have been cases in which deposits were not observed [7]. In addition, the initial cryoglobulin qualitative test showed a borderline positive result; however, cryoglobulin qualitative test results can be affected by temperature control during testing, sample volume, and reaction time of the test [8]. In this case, the amount of cryoglobulin in the blood was small and may not have been a typical result of the cryoglobulin qualitative test and renal biopsy; however, the patient was finally diagnosed with cryoglobulinemic nephritis after other diseases were excluded by performing bone marrow examination and other tests.

Treatment for cryoglobulinemic vasculitis includes treatment of the primary disease, steroids, immunosuppressive drugs, and plasma exchange. Steroid pulses or high-dose systemic glucocorticoids (approximately 1 mg/kg/d) are often administered to treat moderate-to-severe mixed cryoglobulinemic vasculitis. Recently, the therapeutic efficacy of rituximab in combination with these treatments has been reported, and initial treatment with rituximab and steroids is recommended [6]. Several observational studies have also reported improvement in renal function, normalization of urinary sediment, and reduction in proteinuria with rituximab treatment for cryoglobulinemic nephritis [9-12]. Early studies on the treatment of cryoglobulinemic vasculitis and cryoglobulinemic nephritis with rituximab revealed increased remission rates when rituximab was combined with glucocorticoids and immunosuppressive drugs in patients with HCV as a background disease and failed antiviral or immunosuppressive therapy [10,13]. However, there have been reports on the therapeutic efficacy of rituximab alone, without glucocorticoids or immunosuppressive agents, in patients with cryoglobulinemic vasculitis and HCV as a background disease [14,15]. By contrast, for patients with noninfectious mixed cryoglobulinemia without chronic HCV infection, as in this patient, several studies have shown the efficacy of rituximab compared with glucocorticoids alone or a combination of glucocorticoids and rituximab [9,16], but the therapeutic efficacy of rituximab as a single agent has rarely been reported. In a study by Terrier et al. evaluating the efficacy of rituximab in patients with non-infectious cryoglobulinemia, rituximab was administered either as 375 mg/m² weekly for four consecutive weeks or 1000 mg twice, two weeks apart. Combination therapy with corticosteroids demonstrated higher remission rates compared to corticosteroid monotherapy or combination therapy with alkylating agents and corticosteroids, and the prednisone dose could be tapered to less than 10 mg/day after six months [9]. In another report from the same group, although rituximab was used as a first-line therapy in some cases, many patients also received concomitant corticosteroids at a median dose of 30 mg/day or additional immunosuppressive agents such as cyclophosphamide or mycophenolate mofetil. In addition, serious adverse events were reported in patients receiving high-dose corticosteroids with rituximab [16].

Our patient had nonviral cryoglobulinemic nephritis, but because she was a frail older patient who had been using methotrexate and iguratimod for rheumatoid arthritis, she was treated with rituximab alone, without steroids, owing to concerns about infection risk, and she was followed up for over one year. Serum Cr and urinary protein levels decreased, albeit partially. The reduction in complement, which indicates cryoglobulin vasculitis activity, was suppressed, and the cryoglobulin qualitative test yielded negative results. These results indicate that rituximab may be effective as monotherapy for nonviral cryoglobulinemic nephritis. Regarding the safety of rituximab, there are reports that there is a risk of viral reactivation when rituximab is administered in combination with glucocorticoids in patients with HCV background disease compared with glucocorticoid monotherapy [17]. However, there are also reports that rituximab does not increase serious adverse events compared with other immunosuppressive agents or high-dose steroids [13,18]. In this case, there was no risk of reactivation because the patient did not have HCV infection, and no other adverse events were observed during the course of the study. There were concerns that the use of steroids would lead to muscle weakness and a decline in ADL in our patient. However, treatment with rituximab monotherapy did not reduce the patient's ADL, and the patient's daily activities remained the same as before diagnosis.

## Conclusions

In this case, a frail elderly patient with cryoglobulinemic nephritis was treated with rituximab monotherapy without steroids due to concerns about the risk of infection and decreased ADL. As a result, a decrease in serum Cr and urinary protein was observed, and the decrease in complement was also suppressed. In addition, no adverse events or decline in ADL were observed during the course of the study. In terms of efficacy and safety, rituximab should be considered when treating patients with cryoglobulinemic nephritis.
